# Experimental Tests on Fiber-Reinforced Alkali-Activated Concrete Beams Under Flexure: Some Considerations on the Behavior at Ultimate and Serviceability Conditions

**DOI:** 10.3390/ma12203356

**Published:** 2019-10-15

**Authors:** Linda Monfardini, Luca Facconi, Fausto Minelli

**Affiliations:** DICATAM—Department of Civil, Environmental, Architectural Engineering and Mathematics, University of Brescia, 25123 Brescia, Italy; l.monfardini001@unibs.it (L.M.); luca.facconi@unibs.it (L.F.)

**Keywords:** alkali-activated concrete, fly ash, geopolymer concrete, flexure, beams, fiber-reinforced concrete, crack spacing, tension stiffening

## Abstract

Alkali-activated concrete (AAC) is an alternative concrete typology whose innovative feature, compared to ordinary concrete, is represented by the use of fly ash as a total replacement of Portland cement. Fly ash combined with an alkaline solution and cured at high temperature reacts to form a geopolymeric binder. The growing interest in using AACs for structural applications comes from the need of reducing the global demand of Portland cement, whose production is responsible for about 9% of global anthropogenic CO_2_ emissions. Some research studies carried out in the last few years have proved the ability of AAC to replace ordinary Portland cement concrete in different structural applications including the construction of beams and panels. On the contrary, few experimental results concerning the structural effectiveness of fiber-reinforced AAC are currently available. The present paper presents the results of an experimental program carried out to investigate the flexural behavior of full-scale AAC beams reinforced with conventional steel rebars, in combination with fibers uniformly spread within the concrete matrix. The experimental study included two beams containing 25 kg/m^3^ (0.3% in volume) of high-strength steel fibers and two beams reinforced with 3 kg/m^3^ (0.3% in volume) of synthetic fibers. A reference beam not containing fibers was also tested. The discussion of the experimental results focuses on some aspects significant for the structural behavior at ultimate limit states (ULS) and serviceability limit states (SLS). The discussion includes considerations on the flexural capacity and ductility of the test specimens. About the behavior at the SLS, the influence of fiber addition on the tension stiffening mechanism is discussed, together with the evolution of post-cracking stiffness and of the mean crack spacing. The latter is compared with the analytical predictions provided by different formulations developed over the past 40 years and adopted by European standards.

## 1. Introduction

Alkali-activated concrete (AAC) has been studied over the past years as a “green” alternative to ordinary Portland cement (OPC), whose production is energy intensive and responsible for about 8–9% of CO_2_ emissions worldwide [1]. Alkali-activated binders can be generated from different types of aluminosilicate precursors, with differing availability, reactivity, cost, and value worldwide [2]. Because of the need for careful control of formulation, practical difficulties in application and supply chain limitations, geopolymers are still far from a total replacement of OPC across its full range of applications. However, alkali-activated binders may become sustainable and cost-effective construction materials, especially in the case that they are produced using locally-available raw materials [3]. The Australian experience in the field of geopolymers shows that the use of these binders may lead to a potential reduction of 40–60% in greenhouse gas emission, while the financial costs can be even 7% lower compared with OPC [2].

Currently, most literature regarding fly ash alkali-activated concrete focuses mainly on the study of the material properties, whereas limited attention has been paid to the structural behavior of AAC structures. The latter were first investigated by Hardjito et al. (2004) [4], Sumajouw et al. (2005) [5], and Sumajouw & Rangan (2006) [6], who performed a series of flexural tests on reinforced AAC beams and conventional reinforced concrete (RC) beams with different reinforcement ratios (0.64–2.69%). Test results showed a similar behavior of ACC and RC beams in terms of capacity and ductility evaluated for the same reinforcement ratio. A few years later, Dattereya et al. (2011) [7] compared the flexural behavior of reinforced geopolymer and conventional RC beams with reinforcement ratios ranging from 1.82 to 3.33%. According to their results, the normalized ultimate bending moments of all the test beams were quite similar, whereas the normalized bending moment at first cracking of the ACC specimens was generally lower (15–30%) than that observed for the RC beams.

Other studies have been devoted to investigating the ability of existing analytical models, which were originally developed for RC elements made with OPC, to predict the behavior of AAC elements. Yost et al. (2013) [8,9] carried out a series of tests on under-reinforced beams and then applied the models reported by the ACI 318-08 [10] to predict the behavior of the samples at the service and ultimate limit state. The authors concluded that the equations usually used to predict both the elastic behavior and the flexural/shear strength of RC beams can also be applied to get a reasonable estimation of AAC beam responses. Based on different experimental results reported by the literature, Prachasaree et al. (2014) [11] proved the inadequacy of the rectangular stress-block parameters typically used for OPC and proposed new stress-block design equations suitable for ash-based geopolymer concrete. The latter provided a rather good prediction of the flexural response of a series of AAC beams found in the literature.

It is well known that ordinary concrete exhibits brittle behavior because of its low uniaxial tensile strength and mode-I fracture energy. The addition of fibers randomly spread within the concrete matrix is a well acknowledged methodology to improve the tensile strength and toughness of concrete [12,13,14,15]. Experimental and numerical studies recently carried out by Mastali et al. [16] and Kheradmand et al. [17] proved that short hybrid polymeric fibers can be successfully employed to improve the flexural performance of geopolymeric mortar and concrete. As an additional benefit, fibers allow to better control the effects of shrinkage [18], thermal gradients, and any factor determining volumetric instability of the composite material.

The improvement of the tensile behavior of concrete due to the use of fibers can be exploited to enhance the ultimate and the serviceability performance of different kinds of structures [19]. Several research studies have proven the ability of fibers to partially or even totally replace conventional steel reinforcement in structures characterized by a significantly high degree of redundancy, such as slab-on-grade and elevated slabs [20]. Other authors have shown the possibility of partially replacing either the flexural or the shear reinforcement in concrete beams [21]. 

The results of an experimental research performed on full-scale reinforced AAC beams subjected to flexure are herein presented. This study aimed at evaluating the effectiveness of ACC as a structural material in view of its use for the construction of typical pre-cast elements such as ducts, manholes, beams, columns, and roof elements. The experimental program included ACC beams not containing fibers and beams made with ACC reinforced either with steel (rigid) or polymeric (deformed) fibers. The paper will describe and discuss the test results by referring to both the ultimate (ULS) and the serviceability (SLS) loading conditions. Particular attention will be devoted to the analysis of the tension stiffening effect, as well as to the prediction of crack spacing and width according to the analytical models available from different European structural codes and pre-standards. 

## 2. Experimental Program

### 2.1. Properties of the Test Beams

**Figure 1:**
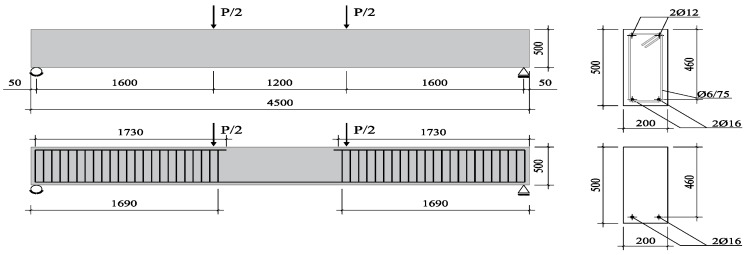
Sample details for full-scale beams, dimensions in millimeters.

A total of five beams were cast and tested in the laboratory of structural engineering of the University of Brescia. As shown in Figure 1, the beams had an overall length of 4500 mm (L), a span length of 4400 mm, and a cross-section of 200 mm (B) × 500 mm (h). Specimens were longitudinally reinforced with two 16 mm diameter (2 Ø16 − A_s,long_ = 402 mm^2^) bottom deformed rebars, resulting in a longitudinal steel ratio (ρ_l_) of 0.43% (concrete clear cover = 26 mm). In the middle portion of the element (in the flexural span between the two point loads), no stirrups and reinforcement bars in compression were provided to promote a flexural collapse governed by concrete crushing. In the remaining portions of the beam, two Ø12 mm bars were provided in the compression zone and closed stirrups (Ø6 mm@75 mm) were placed to prevent shear failure. The main properties of the beams are summarized in Table 1. As one may note, except for the specimen AAC that was cast without fiber reinforcement, the beams SFRAAC-1 and SFRAAC-2 were reinforced with steel (rigid) fibers, whereas the specimens PFRAAC-1 and PFRAAC-2 contained macro-synthetic (deformed) fibers. 

After casting, beams and companion samples for material characterization were left in the wooden molders for 2 days (rest period). At the end of the rest stage, all the specimens were demolded, wrapped with a polyethylene sheet to prevent moisture loss and, finally, they were placed in a climate chamber to undertake curing. During the curing process, the ambient temperature was increased up to 60 °C and then kept constant for 24 h. More details about the adopted curing method are described in [22].

### 2.2. Materials

Steel fiber-reinforced alkali-activated concrete (SFRAAC) and polymeric fiber-reinforced alkali-activated concrete (PFRAAC) were used to cast the specimens SFRAAC-1 and 2 and PFRAAC-1 and 2, respectively. A single control beam made with AAC was cast.

High-strength hook-ended steel fibers, with a length (L_f_) of 30 mm and a diameter (d_f_) of 0.35 mm (aspect ratio L_f_/d_f_ = 85.7), and synthetic embossed macro-fibers with a length of 54 mm and a diameter of 0.80 mm (aspect ratio L_f_/d_f_ = 67.5), were added to the mixture in two different contents (25 and 3 kg/m^3^, respectively, for steel and synthetic fibers) corresponding to a volume fraction (V_f_) of 0.3%. The fiber tensile strength was 2200 MPa (minimum value according to the producer) and 585 MPa, respectively, for steel and synthetic fibers.

The mixture used for casting the AAC beam consisted of coarse aggregate 4–10 mm (1141 kg/m^3^), sand 0–4 mm (615 kg/m^3^), and class F fly ash (472 kg/m^3^) mixed with an alkaline solution composed of 8 M sodium hydroxide (48 kg/m^3^) and sodium silicate (119 kg/m^3^). The chemical composition of the fly ash is reported in Table 2. The silica modulus (i.e., the SiO_2_ to Na_2_O ratio) that characterizes the activator solution was found to be 1.99. Extra water (35 kg/m^3^) was added at the end of mixing just to promote suitable workability. The resulting volumetric mass density (2430 kg/m^3^) of the hardened material was comparable to that of ordinary concrete.

The same mixture adopted for the AAC beam was used to cast the SFRAAC and the PFRAAC beams. In both cases, fibers were added to the mixture at two different times: Half of the total amount of fibers was mixed together with the dry components (i.e., aggregates and fly ash), whereas the remaining part was mixed after the addition of the liquid components (i.e., alkaline solution and extra water). This procedure led to an improved material workability as it allowed an enhanced fiber distribution and prevention of any fiber-balling phenomenon. The volumetric mass density of the hardened material resulted 2455 kg/m^3^ and 2433 kg/m^3^ for the SFRAAC and PFRAAC beams, respectively. 

The mixtures were all characterized by a liquid to fly ash ratio of 0.43, an alkaline solution to fly ash ratio of 0.35, and by a sodium silicate to sodium hydroxide ratio equal to 2.5. The alkaline solution (mixing of sodium silicate and sodium hydroxide) had the same composition and chemical properties of that used in a previous work [23]. It has to be highlighted that extra water had the only aim of improving the workability of the fresh material. This fact explains why water was added some minutes later than the alkaline solution. 

A series of tests were carried out to characterize the mechanical properties of the three materials used in the present research investigation. 

To determine the average cube compressive strength (R_cm_) (see Table 3), uniaxial compression tests were performed on cubes (side = 100 mm) according to EN 12390-3 [24]. Before being tested after 28 days from casting and about 25 days from the end of the curing period, the cubic samples were stored at room temperature of 15–25 °C and relative humidity of 45–60%. As also observed by other authors [2,10,25], the results of the present experimental program [18] show that the compressive strength of the adopted geopolymers stabilizes at the maximum value right after the end of the curing phase, namely 4–5 days after casting. Therefore, the tests performed at 28 days certainly provided the maximum strength of the materials. 

Uniaxial compressive tests on cylinders (height = 200 mm; diameter = 100 mm) were carried out under displacement control in order to determine the Young’s modulus and the compressive stress–strain constitutive law of the materials. Table 3 reports the mean elastic modulus (E_m_), determined according to EN 12390-13 [26], as well as the mean values of the cylindrical compressive strength (f_cm_) and of the corresponding strain (ε_cm_). All the cylinders were tested after 28 days from the casting date. The results show that all the elastic moduli were lower than that (e.g., ~30 GPa) typically exhibited by a traditional OPC-based concrete. As observed by others [2,6,21,27], such a difference can be explained by considering that the C–S–H gel produced by the hydration process of OPC has a higher elastic modulus compared to N–A–S–H gel resulting from the alkaline activation process [28,29]. It also has to be noted that the addition of fibers decreases the workability and introduces air in the matrix, with a consequent possible further reduction of the modulus of elasticity. Conversely, the compressive strain at peak strength (ε_cm_) obtained from the tests on cylinders were about 40–70% higher than the corresponding values reported by the Eurocode 2 [30] (clause 3.1) for OPC concrete with the same mean compressive strengths of the materials tested herein. 

**Figure 2:**
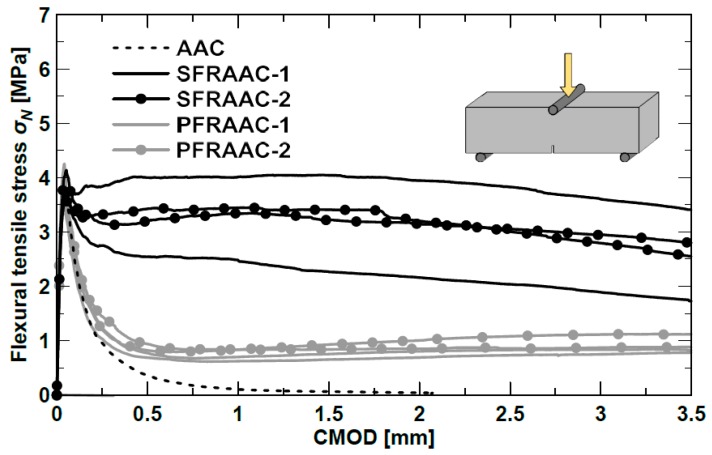
Flexural tensile stress–CMOD curves.

Figure 2 represents the flexural tensile stress vs. CMOD (crack mouth opening displacement) curves of the notched beams (150 × 150 × 500 mm^3^) tested to characterize the tensile post-cracking behavior of the materials. Tests were carried out according to EN 14651-5 [31], which requires the evaluation of the limit of proportionality f_L_ and the residual flexural tensile strengths f_R,1_, f_R,2_, f_R,3_, f_R4_, corresponding, respectively, to CMOD values of 0.5, 1.5, 2.5, and 3.5 mm. As usually observed for OPC concretes, the response of the AAC material not containing fibers was characterized by a significant reduction of the tensile resistance after cracking (i.e., after the achievement of the peak strength) due to the low mode-I fracture energy. Unlike AAC, the material containing steel fibers exhibited a significantly higher post-cracking strength in correspondence of the CMOD values 0.5 mm and 2.5 mm, which are representative of the serviceability and ultimate conditions according fib Model 2010 (MC2010) [32]. Because of the lower tensile performance of the polymeric fibers, the PFRAAC specimens experienced a brittle response, very similar to that observed for the AAC beams except for high values of CMOD, corresponding to residual strengths characterizing ultimate conditions. 

Table 3 summarizes the results from material characterization tests carried out for each of the full-scale test beams. This exhaustive characterization allows a better interpretation of the experimental results described in Section 3.

The longitudinal deformed rebars (B450 C according to Eurocode 2 [29]) were mechanically characterized by testing samples according EN 15630-1 [33]. The characterization tests provided a yielding strength (f_y_) of 535 MPa, an ultimate strength (f_tu_) of 646 MPa, and an ultimate tensile strain of about 13%. 

### 2.3. Test Set-Up and Instrumentation

**Figure 3:**
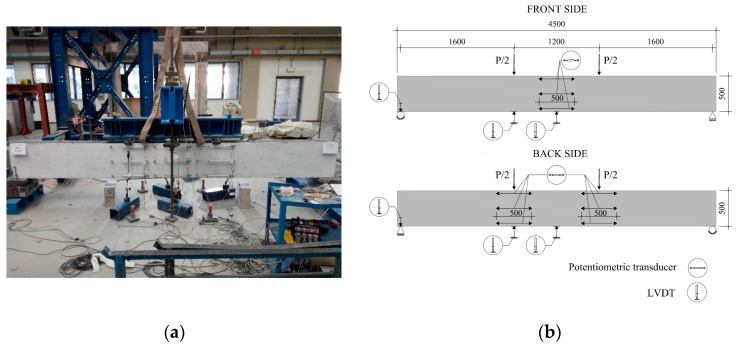
View of the typical 4-point bending test set-up (**a**). Schematic of the instrumentation (dimensions in millimeters) (**b**).

Figure 3a shows the loading set-up adopted to perform the flexural tests on the full-scale beams. The specimen was supported by two steel rollers located at the two ends of the beam (Figure 3b). A couple of steel rollers were also located on the top side of the specimen in order to support the longitudinal spreader steel beam used to apply the two point loads (P/2) acting at a distance of 600 mm from the middle of the beam. To prevent load concentration and possible local failure, each roller was laid on a steel plate positioned on a 25-mm-thick neoprene sheet. The total vertical load (P) was applied to the spreader beam by an electromechanical jack that allowed to perform the test under displacement control.

Figure 3b shows also the typical instrumentation set-up adopted to monitor the specimens. Six linear variable differential transformers (LVDTs) were used to measure deflections at midspan (front and back side) and at supports (front and back side). A total of nine potentiometric transducers were adopted for measuring the horizontal deformations along the height of the cross-sections located at midspan (front side), as well as under the two loading points (back side). In more detail, each cross-section was instrumented by three potentiometers installed, respectively, at 40, 250, and 460 mm from the top of the beam. A load cell was used to monitor the total load applied by the thrust jack. Tests were carried out by monotonically increasing the vertical displacement up to failure. The screw rate of the thrust jack was set at 0.75 mm/min in the initial stage, and then reduced to 0.5 mm/min after rebar yielding.

## 3. Experimental Results and Discussion 

### 3.1. Behavior at Ultimate Limit State (ULS)

**Figure 4:**
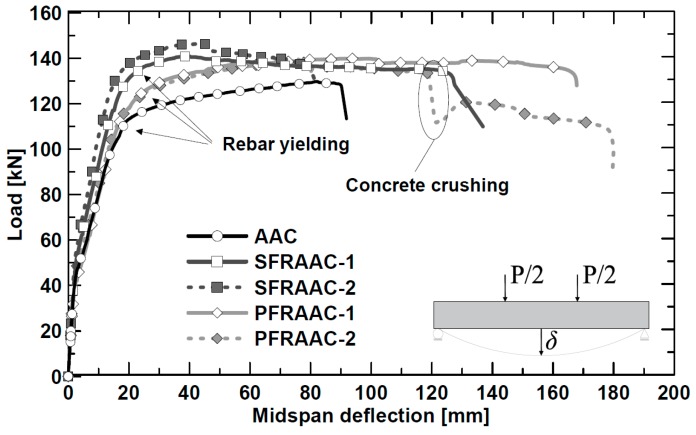
Total load–midspan deflection (δ) curves.

The total load–midspan deflection (δ) curves of the five test beams are depicted in Figure 4. Note that the load reported in the diagram includes both the weight of the loading system and the self-weight of the specimens. 

All beams exhibited the same initial elastic stiffness and similar first cracking loads that preceded the onset of the cracked stage. The kink point at the end of the second branch marks off the limit between the cracked stage and the plastic stage. In the plastic stage (i.e., the third branch), the applied load tended to slowly increase up to the maximum value.

**Figure 5:**
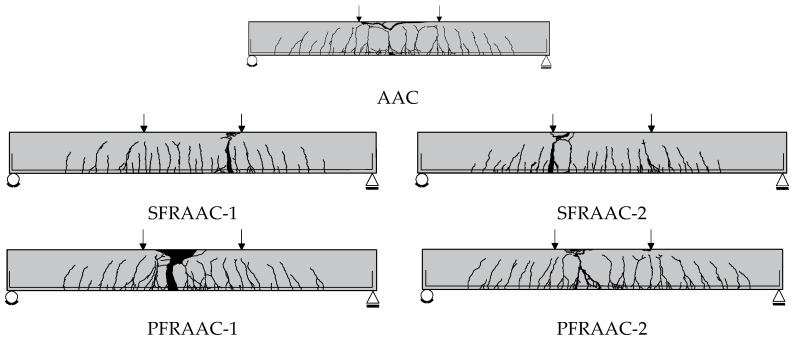
Crack patterns at failure.

As expected, the AAC element achieved the lowest maximum capacity (P_max_ = 130 kN) slightly before the final collapse. The latter occurred in the middle portion of the beam through concrete crushing (Figure 5). 

The maximum capacity (P_max_) of the beams reinforced with steel and synthetic fibers (Table 4) were, respectively, 8–12% and 8% higher than that reached by the AAC specimen. The low longitudinal reinforcement ratio (ρ_l_ = 0.43%) made the contribution of fibers more significant in governing the structural response of the beams. Because of their ability of enhancing concrete toughness in compression, fibers were able to avoid a sudden and brittle crushing of concrete. Moreover, compared to the reference beam, all fiber-reinforced beams experienced a more ductile behavior after yielding, and the final collapse was due to the tensile rupture of the longitudinal reinforcement. In fact, the improved steel-to-AAC interfacial bond [34] prevents or delays the development of plastic deformations in the yielded rebar. This phenomenon leads to a strain localization with a reduction of the portion of the bar under yielding which, with increasing fiber effect, determines an early rebar collapse. 

Beams PFRAAC-1 and PFRAAC-2 presented a slight decrease of the load due to the progression of a rather well controlled concrete crushing mechanism. The development of crushing particularly affected the response of the specimen PFRAAC-1, which exhibited a 15% decrease of the bearing capacity at a midspan deflection of about 120 mm (Figure 4). Figure 5 clearly shows that the significant damage caused by crushing occurred in the central portion of the specimen PFRAAC-1. In spite of concrete crushing, both PFRAAC beams continued to keep a significant load carrying capacity until final rupture of the longitudinal reinforcement occurred. The SFRAAC beams experienced the same failure mechanism, but the sharp reduction of resistance due to crushing was generally not observed. In conclusion, both the SFRAAC and the PFRAAC specimens presented a flexural failure mode characterized by the tensile rupture of longitudinal reinforcement.

**Figure 6:**
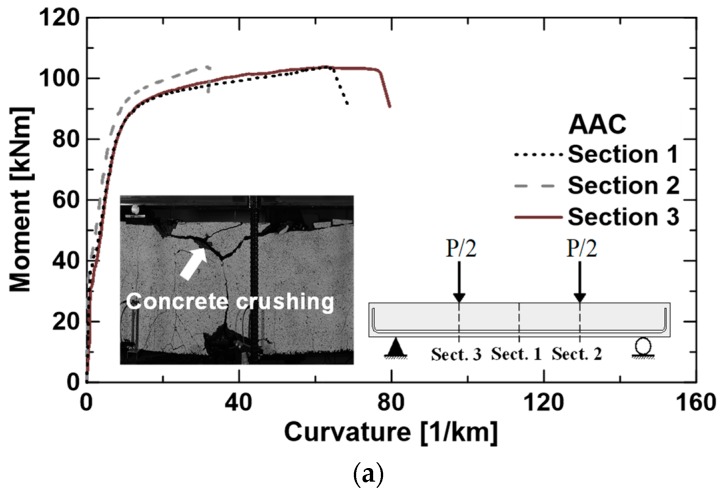

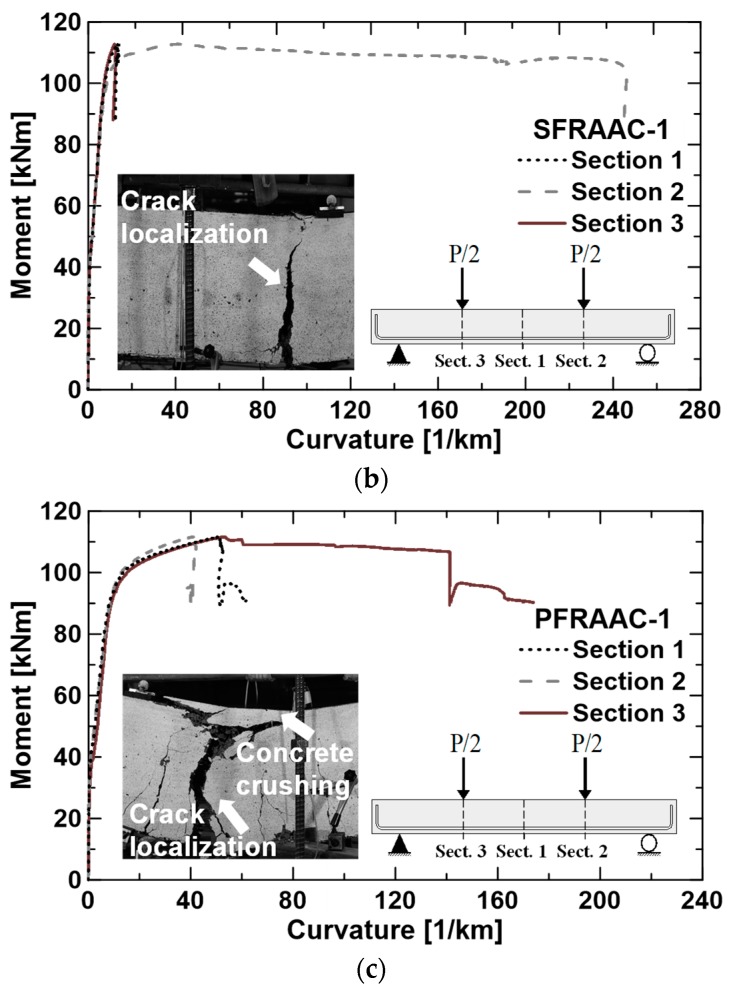
Experimental moment–curvatures curves and pictures of collapse for beams AAC (**a**), SFRAAC-1 (**b**), and PFRAAC-1 (**c**).

The moment–curvature responses detected at different cross-sections, i.e., Section 1 (mid-span) and Section 2 and Section 3 (under the point loads), are potted in Figure 6. Only the curves related to the beams AAC, SFRAAC-1, and PFRAAC-1 are reported, as representative of the typical behavior of each mixture. Based on the classical Navier’s hypotheses, the curvature was calculated by the horizontal deformations measured by the potentiometers installed on the beams. 

The observed responses appeared very similar to those reported by other studies [35] for OPC concrete beams with or without fiber reinforcement.

Moreover, the diagrams allow appreciating the strain localization (i.e., higher deformation) that generally involved one of the three monitored sections in which the ultimate mechanism took place (with significantly high values of local curvature, up to 240 km^−1^, in specimen SFRAAC-1). The strain localization appeared to be more pronounced in the beams containing fibers, where it was promoted by the higher post-cracking tensile strength of the material (Figure 2), which allowed the compression chord to strongly delay its crushing with, conversely, a steady progressive material degradation in compression. Once the strain localization occurred, the beams started to behave like two rigid blocks, able to rotate about both the beam supports and the section subjected to the crack localization (a sort of plastic hinge). 

The small picture reported in Figure 6b shows the flexural collapse mode of the SFRAAC-1 beam, which was not affected by significant damage of the top side of the cross-section. On the contrary, the picture of Figure 6c illustrates a high level of strain both at the bottom (steel strain) and at the top chord (concrete crushing). They both concurred in determining the collapse of the specimen PFRAAC-1, which experienced rebar collapse in the end.

**Figure 7:**
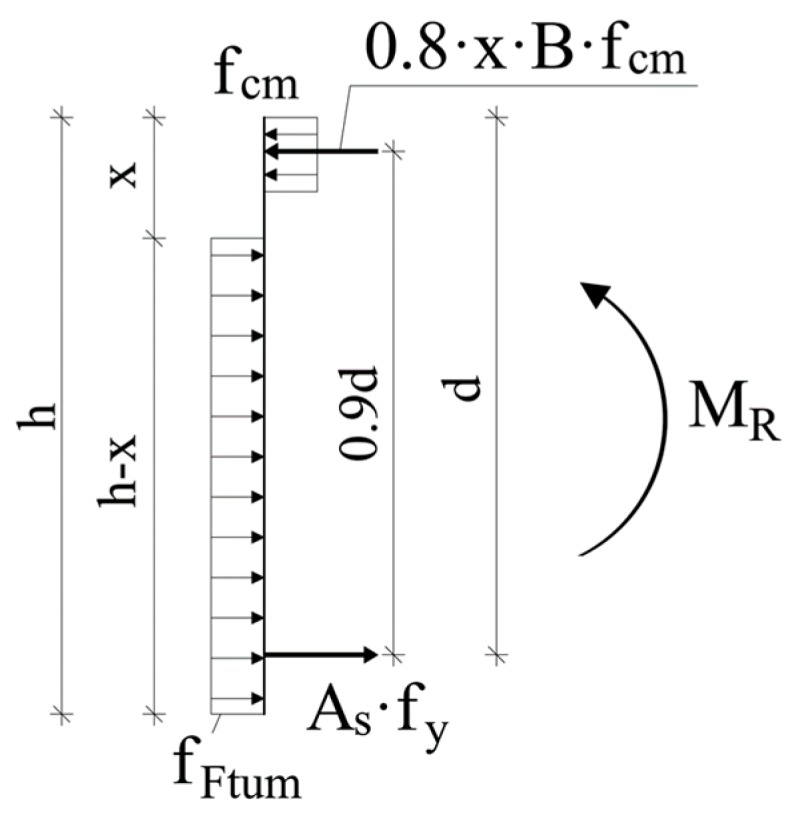
Simplified stress-block model for flexural strength calculation according MC2010 [33].

In order to predict the flexural resistance of the test beams, the simplified rectangular stress-block model schematized in Figure 7 can be adopted. It is seen that for fiber-reinforced beams, the tensile resistance of the material is considered by means of the rigid-plastic model reported by the MC2010 (clause 5.6.4) [33], which considers a constant residual tensile strength (*f_Ftu_*) over the depth *(h-x)* of the cross-section. Based on the previous assumptions, by also supposing that tensile reinforcement is yielded (reasonable assumption considering the low reinforcement ratio selected), the resisting moment (*M_R,max,anl_*) can be calculated as follows:

(1) $$M_{R,max,anl}{=f}_{y}\cdot A_{s}\cdot \left(d-0.4\cdot x\right){+f}_{Ftu}\cdot B\cdot \left(h-x\right)\cdot \left(0.5\cdot h+0.1\cdot x\right)$$

where *f_Ftu_* = *f_R3_*/3 is the tensile strength of fiber-reinforced concrete; *f_R3k_* is the residual flexural strength at a crack mouth opening displacement (CMOD) of 2.5 mm (see Table 3); *h* = 500 mm is the section height; *B* = 200 mm is the section width; *A_s_* = 400 mm^2^ is the total area of bottom longitudinal rebars; *f_y_* = 535 MPa is the yielding strength of rebars; and *d* = 460 mm is the effective depth. From the equilibrium of horizontal forces, the neutral axis depth (x) is obtained:

(2) $$x=\frac{f_{y}\cdot A_{s}{+f}_{Ftu}\cdot B\cdot h}{B(0.8\cdot f_{cm}{+f}_{Ftu})}$$

in which *f_cm_* is the cylindrical compressive strength of concrete, as reported in Table 3. The results reported in Table 4 show that the resisting moment obtained from Equation (1) slightly underestimates (−9%) the maximum experimental bending moment (*M_R,max,exp_*). On the contrary, the predicted bending moments related to beams containing steel fibers were basically equal or slightly higher (+2%) than the experimental ones. The analytical model confirmed the minor contribution provided by fibers to the flexural resistance of the beams. However, the simplified cross-sectional model proved to be able to well predict the resistance of all the AAC beams tested in this research study, in contrast to what is stated in [11].

Table 4 also reports the ductility indexes evaluated as the ratio between the ultimate (Ø_u_) and the yielding (Ø_y_) curvature (μ_Ø_ = Ø_u_/Ø_y_), as well as the ratio between the ultimate (δ_u_) and the yielding (δ_y_) midspan deflections (μ_δ_ = δ_u_/δ_y_). Irrespective of the ductility index considered, FRAAC beams resulted to be more ductile than the reference specimen. When considering the ductility in terms of curvature, the average ductility of the SFRAAC beams appears to be higher than that observed for the specimen containing synthetic fibers. Contrariwise, whether the ductility is assessed by the index, which better represents the overall response of the specimen instead of the behavior of the single local cross-section, the ductility of the beams containing synthetic fibers results to be the highest. In fact, once strain localization occurred, the lower post-cracking resistance of concrete reinforced with synthetic fibers allowed to delay the rebar failure, thus promoting a higher ductility (+60%) than that exhibited by the SFRAAC specimens. 

It seems that, in flexural elements with low reinforcement ratios, synthetic deformed fibers are able to postpone (compared to steel fibers) the strain localization at the rebar level; in addition, their influence on the compression chord, even though less pronounced compared to steel fibers, allows anyway a progressive and controlled decay of the compression chord resistance. The combination of these two effects is beneficial in terms of overall structural ductility (i.e., ductility on terms of displacement). However, this trend should be cautiously evaluated by testing different longitudinal reinforcement ratios.

### 3.2. Behavior at Serviceability Limit State (SLS)

The change of stiffness occurring between the first and the second branch of the curves, shown in Figure 4, defines the onset of the cracking stage. All of the beams cracked at a load of about 45 kN, which corresponded to a midspan deflection approximately equal to δ_cr_ = 1.7 mm. Considering that the materials used in the present investigation had basically the same peak tensile flexural strength (f_L_) (see Table 3), similar first cracking loads were expected.

Crack control is one of the main features that must be considered in designing concrete members under service loading conditions. To estimate the mean crack width (w) in a concrete member, the following general equation can be used:

(3) $${w=S}_{rm}\cdot \left(\varepsilon_{sm}{-\varepsilon }_{cm}\right)$$

where *ε_sm_* is the mean tensile strain in the reinforcement; *ε_cm_* is the mean tensile strain in concrete between cracks; and *S_rm_* is the mean crack spacing. A reasonable prediction of crack width necessarily requires the estimation of the crack spacing parameter.

**Figure 8:**
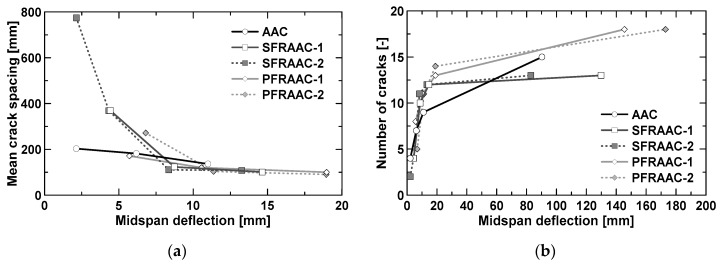
Mean crack spacing in the pre-yielding phase (**a**) and number of cracks (**b**) vs. midspan deflection.

The results of the tests performed herein allowed to calculate the crack spacing as the mean distance between cracks, namely the distance between the point loads (i.e., 1200 mm) divided by the number of cracks detected in the constant bending moment region. Figure 8a reports the evolution of the mean crack spacing observed in the pre-yielding stage against the deflection at midspan. The evolution of the number of cracks detected during the execution of each bending test is represented in Figure 8b. 

As usually observed in OPC concrete members, the mean crack spacing tended to decrease with increasing vertical deflection and then stabilized at a minimum value (stabilizing cracking stage). The crack spacing became almost constant at a deflection of about 8 mm, which corresponded to a vertical load equal to 60% (*p* ≈ 80 kN) of the maximum capacity. The attainment of a constant crack spacing represents the onset of the so-called stabilized cracking stage. The crack spacing detected in the stabilized cracking stage for the fiber-reinforced beams was similar or slightly lower (−10%) than that presented by the reference beam not containing fibers. On the contrary, for vertical deflections ranging from first cracking (δ_cr_ = 1.7 mm) to 7.5 mm, the crack spacing of the reference beam resulted to be the lowest, together with that presented by beam PFRAAC-1. This result appears to be in contrast to what is reported by other authors [36,37], especially with regard to the specimens made with steel fiber-reinforced concrete. Because of its higher post-cracking strength, fibers are usually able to reduce the crack spacing even if provided in low (<0.5%) volume fractions. Further investigation needs to be undertaken to better understand these experimental observations.

As the crack spacing decreased in the pre-yielding stage, the number of cracks (Figure 8b) increased at a very high rate. After yielding, the formation of new cracks still took place, but at a slower rate compared to the pre-yielding stage. It is worth noting that the beams characterized by the lowest number of cracks at failure were those containing steel fibers (Figure 8b). This fact can be explained by considering the ability of steel fibers to localize the deformation in a single section, as already shown in the paragraph related to the behavior at ultimate limit states. 

To estimate the mean crack spacing for RC members, European structural codes have proposed different relationships over the past years. The CEB-FIP Model Code 1978 [38] proposed the following equation:

(4) $$S_{rm}=2\cdot \left(c+\frac{s}{10}\right){+k}_{1}\cdot k_{2}\cdot \frac{\emptyset }{\rho_{eff}}$$

where:

*c* = concrete clear cover;

*s* = distance between longitudinal reinforcement bars;

*k*_1_ = coefficient regarding bond between bars and concrete (=0.4 for deformed bars);

*k*_2_ = coefficient regarding stress distribution in the cross-section (=0.125 for flexure);

*Ø* = longitudinal rebar diameter; and

*ρ_eff_* = effective reinforcement ratio.

In 1990, the updated version of the same European code (CEB-FIP Model Code 1990 [39]) proposed a simplified formulation depending only on the effective reinforcement ratio and the bar diameter, as follows:

(5) $$S_{rm}=\frac{2}{3}\cdot \frac{\emptyset }{3.6\cdot \rho_{eff}}$$

The first version of the Eurocode 2 published in 1991 [40] reported a modified version of the CEB-FIP Model Code 1978’s [39] model:

(6) $$S_{rm}=50+0.25\cdot k_{1}\cdot k_{2}\cdot \frac{\emptyset }{\rho_{eff}}$$

in which *k_1_* and *k_2_* can be assumed equal to 0.8 (deformed bars) and 0.5 (members in flexure), respectively. The Eurocode 2 released in 2003 [41] re-introduced the clear cover as a parameter affecting the crack spacing:

(7) $$S_{rm}=3.4c+0.425\cdot k_{1}\cdot k_{2}\cdot \frac{\emptyset }{\rho_{eff}}$$

Finally, the MC2010 [33] defined the crack spacing as a function of the transition length (l_s,max_) according to the following relation:

(8) $$S_{rm}=1.17\cdot l_{s,max}=k\cdot c+0.25\cdot \frac{f_{ctm}}{\tau_{bm}}\cdot \frac{\emptyset }{\rho_{eff}}$$

where *f_ctm_* is the mean tensile strength of concrete (Table 3) and *τ_bm_* = 1.8 *f_ctm_* is the mean value of bond stress between concrete and rebars.

Regarding steel fiber-reinforced concrete elements, one of the earliest models for predicting the crack spacing was proposed by the RILEM committee TC 162-TDF (2003) [42], which modified the Eurocode 2 (1991)’s relation [41] for plain concrete as follows:

(9) $$S_{rm}=\left(50+0.25\cdot k_{1}\cdot k_{2}\cdot \frac{\emptyset }{\rho_{eff}}\right)\cdot \frac{50}{L_{f}/d_{f}}$$

Note that in the previous equation, the effect of fibers was considered by the factor 50/(*L_f_/d_f_*) ≤ 1, which includes the fiber aspect ratio *L_f_/d_f_*. Despite the fact that this formulation was initially meant only for elements reinforced with steel fibers, it will be here applied also to the specimens containing synthetic fibers. 

The latest model for predicting the crack spacing in fiber-reinforced concrete elements was reported by the MC2010 (2013) [33]. The latter adjusted the formulation originally developed for RC concrete as follows:

(10) $$S_{rm}=1.17\cdot l_{s,max}=k\cdot c+0.25\cdot \frac{\left(f_{ctm}{-f}_{Ftsm}\right)}{\tau_{bm}}\cdot \frac{\emptyset }{\rho_{eff}}$$

It is seen that the ability of fibers to reduce the crack spacing is considered by the term *f_Ftsm_* = 0.45 f_R1m_, which is related to the residual strength of concrete (*f_R1m_*) corresponding to a crack width (CMOD) of 0.5 mm significant for serviceability loading conditions [33]. 

**Figure 9:**
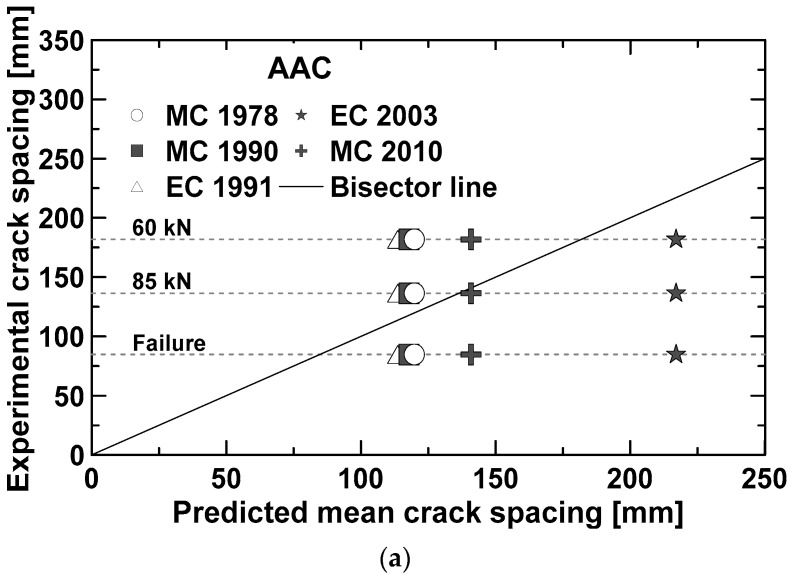

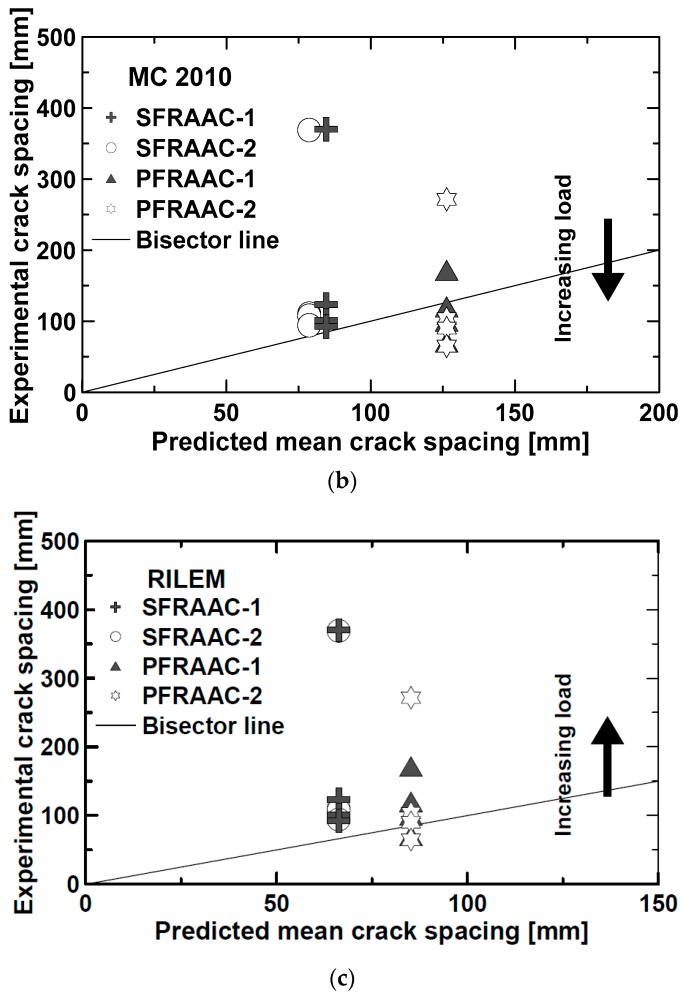
Comparison between predicted and experimental crack spacing. AAC beam (**a**). Predictions according MC2010 [33] (**b**) and RILEM [42] (**c**) for the beams reinforced with fibers.

The diagrams of Figure 9 compare the experimental mean crack spacings, detected at different loading levels (i.e., 60 kN, 85 kN, 115 kN, failure load), with the corresponding values predicted by the models described above. From simple calculations, the tensile stress expected in the longitudinal reinforcement at the minimum loading level (i.e., 60 kN) is about 260 MPa. The latter is generally considered as the maximum value for the adopted reinforcing steel able to limit deflections and ensure good crack control at SLS conditions. It is worth noting that 60 kN and 85 kN correspond, respectively, to 40–46% and 60–65% of the maximum load attained by the test beams.

Regarding the AAC beam (Figure 9a), a good agreement between experimental and analytical data was observed except for the Eurocode 2 (2003) [42], whose predictions overestimated the experimental results both for low and high values of the applied load. 

About the beams reinforced with steel fibers, both RILEM TC 162-TDF (2003) [42] and MC2010 [33] provided crack spacings 30–60% lower than those detected in the stabilized cracking stage (i.e., *p* ≥ 85 kN). When considering the specimens containing synthetic fibers, the prediction of RILEM TC 162-TDF (2003) (Figure 9c) appeared to be very close to the experimental results. On the contrary, the MC2010 (Figure 9b) tended to overestimate by about 20% the stabilized value of crack spacing. 

The effect of fibers on tension stiffening can be highlighted by comparing midspan deflection of the fiber reinforced concrete beams (see Figure 4) with those exhibited by the reference beam (AAC) at a certain loading level. Here, the comparison was carried out by considering three different loading levels, i.e., 40 kN, 58 kN, and 90 kN, which corresponded, respectively, to 30%, 45%, and 70% of the maximum capacity (P_max_ = 130 kN) of the reference beam. The resulting relative variation of midspan deflections will be here referred to as Δ_0.30_ (*p* = 40 kN), Δ_0.45_ (*p* = 58 kN), and Δ_0.70_ (*p* = 90 kN) (Table 5). As shown in Table 5, both SFRAAC beams were characterized by a remarkable increment of the flexural post-cracking stiffness that, in turn, led to a reduction of the maximum deflection ranging from 16% to 40%. Compared to steel fibers, the synthetic fibers resulted to be less effective as the deflection reduction was approximately equal to 7% for the beam PFRAAC-2, and 11–17% for the specimen PFRAAC-1. 

This enhanced post-cracking stiffness, related to low level of strains in the rebars (for the same load levels), is a key factor determining a decrease of the crack widths in flexure, confirming several findings in the literature [37]. 

## 4. Concluding Remarks 

Experimental results on full-scale beams made of AAC under flexure were presented and discussed in this paper, focusing on the structural response and fiber influence on the global and local behavior.

It was observed that the post-cracking response of FRAACs is the most influencing parameter for the structural behavior of beams herein discussed. For this particular geometry and longitudinal steel ratio, fibers promoted enhancements both at SLS and ULS conditions.

Based on the results herein presented and discussed, the main following conclusions can be drawn:

- For the studied element geometry and longitudinal reinforcement ratio (*ρ_l_* = 0.44%), the presence of fibers affected the structural failure mode by promoting a collapse due to rebar rupture; on the contrary, the reference element without fibers (AAC) experienced a classical failure due to concrete crushing.
- The post-cracking strength of fibers, at ULS, developed a strain localization with a reduction of the portion of the bar under yielding. This determined an early rebar collapse, especially for steel fibers, which are more rigid and tougher compared to synthetic fibers. This peculiarity promoted a quite different local behavior well captured by the local moment–curvature diagrams observed: The section where the collapse occurred experienced a greater curvature, resulting in a corresponding larger local ductility, especially for FRC elements with higher post-cracking strengths (steel fibers). Conversely, the lower post-cracking strength observed in polymeric fibers tended to delay, in terms of midspan deformation, the final rebar collapse, resulting in a greater overall ductility (in terms of displacement), also possible thanks to the fiber ability to effectively promote a progressive decay of the concrete in compression.
- Elements reinforced with steel fibers developed the lowest number of cracks at ULS, due to the strain localization, which might have caused a limited (not full) development of crack spacing.
- The crack stabilizing stage for elements reinforced with fibers took place at a higher load level compared to non-fibrous elements; at the crack stabilizing stage, a good agreement between analytical and experimental crack spacing values was observed.
- At SLS conditions, despite AAC beams experiencing a greater elastic modulus, fibers promoted an enhancement of the post-cracking stiffness with a consequent reduction of the midspan displacement and crack widths; a tension stiffening effect was seen to be more noticeable in elements reinforced with steel fibers where post-cracking strengths were greater.

## Figures and Tables

**Table 1:** Main properties of the test beams.

Specimen Designation	B	h	L	ρ_l_	Type of Fibers	Fiber Content	Fiber Volume Fraction (V_f_)
	[mm]	[mm]	[mm]	[%]	[-]	[kg/m^3^]	[%]
AAC	200	460	4500	0.44	No fibers	-	-
SFRAAC-1					Hooked-end steel	25	0.3
SFRAAC-2							
PFRAAC-1					Synthetic Macrofiber—Embossed	3	0.3
PFRAAC-2							

**Table 2:** Chemical composition of fly ash.

Al_2_O_3_	SiO_2_ *	CaO	Fe_2_O_3_	MgO	K_2_O	Na_2_O	TiO_2_	SO_3_
(%)	(%)	(%)	(%)	(%)	(%)	(%)	(%)	(%)
28	56	2	5.5	0.2 ÷ 3	0.2 ÷ 2	0.1 ÷ 0.6	0.1 ÷ 1.7	0.2 ÷ 2

* 40% is composed by reactive silica, representative of pozzolanic potential of fly ash.

**Table 3:** Mechanical properties of materials.

Property	Unit	Test Beams				
		AAC	SFRAAC-1	SFRAAC-2	PFRAAC-1	PFRAAC-2
***E_m_***	[GPa]	23.5 (4.0%)	18.2 (3.0%)	18.7 (1.0%)	18.1 (1.0%)	18.6 (2.0%)
***ε_cm_***	[‰]	3.1 (1.8%)	5.0 (6.0%)	3.4 (12.8%)	2.8 *	2.8 *
***R_cm_***	[MPa]	37.0 (4.1%)	45.0 (2.2%)	34.0 (8.5%)	40.0 (2.0%)	36.0 (3.3%)
***f_cm_***	[MPa]	37.0 (1.0%)	27.0 (0.4%)	24.0 (2.9%)	24.0 (11.0%)	24.0 (11.0%)
***R_cm_/f_cm_***	[-]	1.0	1.7	1.4	1.7	1.5
***f_ck_***	[MPa]	34.5	25.5	20.9	22.8	22.7
***f_ctm_***	[MPa]	3.2	2.6	2.3	2.4	2.4
***f_L_***	[MPa]	3.6 *	3.6 (0.6%)	3.9 (5.7%)	3.8 (15.7%)	3.6 (6.0%)
***f_R_*_1_**	[MPa]	0.37 *	3.3 (31.5%)	3.3 (2.4%)	0.7 (4.0%)	0.9 (4.7%)
***f_R_*_2_**	[MPa]	-	3.2 (39.8%)	3.3 (2.0%)	0.7 (5.0%)	0.9 (3.5%)
***f_R_*_3_**	[MPa]	-	2.9 (43.2%)	3.0 (0.6%)	0.8 (4.2%)	1.0 (8.1%)
***f_R_*_4_**	[MPa]	-	2.6 (46.3%)	2.7 (3.3%)	0.8 (1.9%)	1.0 (8.3%)

Coefficient of variation in round brackets; *f_ctm_ = 0.3·(f_ck_)^2/3^* according Eurocode 2 (2005); *f_ck_* = characteristic value of the cylindrical compressive strength; * only one sample available for the test beam.

**Table 4:** Behavior at ultimate limit states (ULS): Main test results and resisting moment obtained from the simplified cross-sectional model.

Specimen	P_max_	M_R,max,exp_	x	M_R,max,anl_	Ø_u_	Ø_y_	*μ_Ø_*	*δ_u_*	*δ_y_*	*μ_δ_*
	[kN]	[kNm]	[mm]	[kNm]	[1/km]	[1/km]	[-]	[mm]	[mm]	[-]
AAC	130	104	36.1	95	80	12	6.67	92	20	4.65
SFRAAC-1	141	113	59.4	116	246	18	13.67	132	19	6.96
SFRAAC-2	146	117	59.4	116	139	16	8.69	93	17	5.49
PFRAAC-1	140	112	43.8	102	175	20	8.75	194	18	10.8
PFRAAC-2	140	112	43.8	102	174	26	6.69	173	19	9.1

**Table 5:** Behavior at serviceability limit states (SLS): Relative variation of midspan deflection referred to the beam AAC.

Specimen	Δ_0.30_	Δ_0.45_	Δ_0.70_
	[mm]	[mm]	[mm]
AAC	-	-	-
SFRAAC-1	−0.32 (−16%)	−1.84 (−35%)	−3.14 (−26%)
SFRAAC-2	−0.36 (−17%)	−2.11 (−40%)	−3.12 (−26%)
PFRAAC-1	−0.34 (−17%)	−0.67 (−13%)	−1.64 (−11%)
PFRAAC-2	−0.03 (−1.2%)	−0.37 (−7%)	−0.80 (−7%)
